# Enhancing positive memory schemas with tDCS: a pilot study

**DOI:** 10.3389/fnhum.2026.1722485

**Published:** 2026-03-16

**Authors:** Robbert S. Baxendell, Angeliki Sideri, Jan Spijker, Ger P. J. Keijsers, Indira Tendolkar, Janna N. Vrijsen

**Affiliations:** 1 Behavioural Science Institute, Radboud University, Nijmegen, Netherlands; 2 Expert Center for Depression, Pro Persona Mental Health Care, Nijmegen, Netherlands; 3 Department of Psychiatry, Donders Institute for Brain, Cognition and Behaviour, Radboud University Medical Center, Nijmegen, Netherlands; 4 Department of Clinical Psychological Science, Maastricht University, Maastricht, Netherlands

**Keywords:** dorsolateral prefrontal cortex (DLPFC), dysphoria, memory bias, positive mood induction, schema, tDCS

## Abstract

**Background:**

Negative schemas during depression drive persistent memory biases. Positive mood induction procedures (MIPs) can briefly counteract these but are often short-lived. Non-invasive brain stimulation (NIBS) may enhance MIPs. We tested whether anodal transcranial direct current stimulation (tDCS) over the left dorsolateral prefrontal cortex (DLPFC), compared to sham tDCS, could strengthen and prolong MIPs effects on mood and resulted in weaker negative memory bias.

**Methods:**

For this sham-controlled, within subject counterbalanced study we selected 20 dysphoric participants (BDI-II > 13). Mood state was assessed via visual analogue scales before and immediately after the MIP and at session end. Memory was assessed using the Deese-Roediger-McDermott (DRM) false-memory task and a Self-Referent Encoding Task, measuring recall accuracy, recognition indices (hits, false alarms, d′), and self-referent memory bias.

**Results:**

Condition and time did not interact on mood states in this pilot study. However, exploratory analyses revealed that active tDCS significantly reduced sadness at the final time point compared to sham tDCS. The active tDCS condition resulted in less false recognition of negative words in the DRM Task, but the condition and valence interaction were non-significant for hit rates. Participants in the active tDCS condition better discriminated against critical lures and recalled more words overall.

**Conclusion:**

tDCS over the DLPFC may prolong MIP effects, reduce negative memory bias and promote more positive, mood-congruent schema processing. These findings support integrating NIBS with personalised MIPs as a promising avenue for decreasing depression characteristics. Future work should test repeated tDCS sessions, larger samples, and ecologically valid memory bias and mood assessments to strengthen clinical relevance.

## Introduction

Schemas are associative knowledge structures that impact information processing when activated (Richter et al., 2019). They play a fundamental role in memory processing (Gilboa and Marlatte, 2017) and mental disorders (Taylor et al., 2017; Young et al., 2003). Schemas are mostly formed during childhood and serve as a framework through which information is encoded, consolidated, and retrieved. By favouring congruent information, schemas facilitate the assimilation of new information into existing memory schemas (Fernández and Morris, 2018; LaBar and Cabeza, 2006; Van Kesteren et al., 2012). Schema congruent processes are central to the (neuro-)cognitive model of depression (Beck, 2008; Disner et al., 2011), with self-referential information processing playing a particularly influential role (Everaert et al., 2022). This form of cognition involves self-devaluating thoughts that reinforce negative schemas, thereby promoting further negatively biased memory encoding and retrieval (Gotlib and Joormann, 2010; Mathews and MacLeod, 2005).

Negative memory biases, an automatic preference for negative over positive information, are dominant in depression and reinforce maladaptive thinking and depressive symptoms (Beck and Bredemeier, 2016; Everaert et al., 2022; Fleurkens et al., 2025; LeMoult et al., 2016; LeMoult and Gotlib, 2019). Although schemas are generally stable, mood can activate them and intensify negative memory biases through mood-congruent recall, linked closely to Bower’s network theory (Baxendell et al., 2025; Bower, 1981; Gotlib and Joormann, 2010; Howe and Malone, 2011; Richter et al., 2019). Emotional state-dependence is central to Bower’s theory, which holds that current mood activates mood-congruent nodes in semantic memory, leading to mood-congruent recall (Faul and LaBar, 2022). Enhancing methods to counter schema congruent negative memory tendencies in depression through mood-congruent recall therefore provides a foundation for more effective therapeutic interventions.

Mood induction procedures (MIPs) are eminently suited to examine the causal effects of mood on schema memory and memory biases. These procedures provide a controlled way to temporarily elicit or enhance affective states, creating a reliable form of emotional state dependence (Gillies and Dozois, 2021; Marcusson-Clavertz et al., 2019). Among various MIPs, those using autobiographical stimuli and/ or personalised approaches have gained increasing attention, with multi-modal MIPs (e.g., combined music, verbal, and/ or visual cues) proving particularly effective at inducing mood (Fernández-Pérez et al., 2022). In a meta-analysis (Joseph et al., 2020), MIPs are shown as consistently effective at inducing specific mood states, although these effects tend to be short-lived, especially in studies including depressed individuals (Besten et al., 2023; Horner et al., 2014; Kuijsters et al., 2016). Gillies and Dozois (2021) emphasise the limitations of short induction effects in MIPs and suggest exploring enhancement techniques.

Brain states can also be influenced by neuromodulation techniques, which may enhance MIPs. Specifically, transcranial direct current stimulation (tDCS; Brunoni et al., 2016) is of interest here, as it is low-cost and non-invasive with potential for at-home application (Charvet et al., 2020). In tDCS, low-amplitude electrical currents are applied to the scalp to modulate the resting potential of neurons. When applied to the dorsolateral prefrontal cortex (DLPFC), a region central to cognitive control and emotion regulation, tDCS enhances cortical excitability and synaptic plasticity (Brunoni et al., 2016; Liu et al., 2023; Palm et al., 2012; Rahman et al., 2013). In turn, this downregulating of the subcortical structures such as the amygdala yield dampened emotional reactivity (Kensinger and Ford, 2021). Some literature also suggests brief tDCS sessions can enhance memory performance in general (Javadi et al., 2012) and improve cognitive control in depressed individuals specifically (Wolkenstein and Plewnia, 2013). By enhancing the DLPFC function, tDCS might facilitate more adaptive (mood-incongruent) schema processing and decrease the intrusion of (mood-congruent) negatively valanced memories during processing of emotional information. This aligns with its clinical benefits in treatments for mood and anxiety disorders (Ciullo et al., 2021; Kricheldorff et al., 2022; Segrave et al., 2014; Stein et al., 2020) and its small trans-diagnostic effect on working memory and improved attention/vigilance across diagnoses (Begemann et al., 2020).

Combining prefrontal neuromodulation with positive MIPs reduces false recognition of negative schema-congruent material, providing experimental support for a causal role of the prefrontal cortex (PFC; Bovy et al., 2020), and specifically the DLPFC (Brunoni et al., 2014; Morgan et al., 2014). Complementary to this, De Raedt et al. (2017) showed the anodal modulation of the DLPFC with tDCS can lead to more potent positive schemas, which in turn enhanced positive emotional memory in dysphoric individuals. This finding supports the potential of DLPFC stimulation to modulate mood-congruent memory. While previous studies have shown that neuromodulation can alter negative memory biases in individuals with subclinical depression, the combination of tDCS with a positive self-referential MIPs has not yet been examined in relation to both memory and mood duration in clinically relevant populations. The current experiment aimed to pilot whether tDCS coupled with positive MIPs can enhance positive memory biases and induced mood duration and how strong these effects are. To that end, we examined the effect of anodal versus sham tDCS on both implicit and explicit emotional schema-based memory in dysphoric individuals. Additionally, we assessed the duration of mood changes and self-relevant memory biases during the experiment. Both research questions were tested using established tasks, namely the Deese–Roediger–McDermott False Memory Task (DRM Task; Roediger and Mcdermott, 1995) and the Self-Referent Encoding Task (SRET; Derry and Kuiper, 1981). We hypothesised that compared to a sham tDCS session, an active tDCS session during a self-relevant positive MIPs enhances and prolongs mood benefits and yields improved memory performance, particularly by reinforcing positive memory bias and weakening negative memory bias. Primary and secondary analyses testing these hypotheses were preregistered, while additional analyses examining valence-specific effects and related outcomes were prespecified as exploratory to inform future research. The pre-registered hypotheses were: 1. tDCS combined with positive schema activation facilitates the retrieval of positive information in dysphoric individuals. 2. tDCS combined with positive schema activation results in a higher average positive and lower negative mood ratings, increased memory performance and a positive memory bias.

## Methods

### Participants

Participants were approached in-person and through online advertisement after which they were screened for eligibility using the safety screening for tDCS and the BDI-II. All participants were informed both verbally and in writing following proper informed consent procedures. For this pilot study, 166 people were pre-screened, out of which, a sample of 24 adult dysphoric individuals [based on the Beck Depression Inventory second edition, BDI-II, scores > 13; (Beck et al., 1996; Van der Does, 2002)] were recruited and randomised. While four participants failed to complete both sessions due to drop-out, there were no instances of withdrawn consent. The forthcoming analysis constitutes only of the data from the 20 participants (*M* age: 28; SD 10.01, *M* BDI-II score: 24; SD 9.96, 12 Female) who completed both sessions.

### Session outline and interventions

The participants completed the two sessions at Radboud University Medical Centre scheduled 7–14 days apart. Each participant underwent active and sham tDCS sessions paired with a positive MIP in a randomised condition and counterbalanced session within-subject design. After inclusion, each participant was randomly allocated using an online research randomiser to the order of stimulation condition (1st session active and 2nd session sham tDCS, and vice versa), and to the version of the tasks. There were two versions of each task; one for each session, in order to minimise learning effects.

The sessions began with a tDCS safety check and informed consent, followed by an active- (2 mA for 20 min per session) or sham tDCS intervention depending on session randomisation. For this intervention, CE-approved equipment was used with anode placement over the left DLPFC (F3) and cathode over the right supraorbital area (FP2), following 10–20 system guidelines and using conductive rubber electrodes (35 cm^2^, ±0.03 mA/cm^2^) with saline-soaked sponges. The electrode placement was performed using the Beam F3 System (Beam et al., 2009). Both conditions included 10-s ramping, with participants reporting minimal discomfort (with one experiencing light skin irritation which was noted as an adverse event) from standardised, guideline-supported procedures. Participants were blinded to their condition in each session, however due to the version of the tDCS apparatus used (DC-Stimulator Plus, neuroConn GmbH, Ilmenau, Germany) the experimenters performing the stimulation were not blinded to the condition since that option is not offered. Blinding success of participants was assessed at the end of the second session by asking them if they felt there was a difference between the two sessions. After considering their answers, it was evaluated that even though some participants did recognise a difference in the sensation of the stimulation, they did not acknowledge a “placebo” condition, and it was thus deemed that the data were not affected because of failed blinding.

During the tDCS intervention, a multimodal positive MIP was performed. In the first part, participants listened to 5 min of happy music that they had prepared themselves. Prior to the session, participants were instructed to create a playlist in their own device (mobile phone, MP3) which should be at least 5 min in duration and include fun music to get into a good mood. That playlist was used for both sessions with the same sequence of songs. The second part consisted of an adapted version of the Autobiographical Memory Task (AMT, Appendix 1; Raes et al., 2009) where participants were given 5 positive stimuli words and were instructed to vividly recall a personal memory related to that word. To ensure that the participants had understood the task and were recalling the memories as vividly as possible, two practice positive stimulus words were used prior to the five actual stimulus words. After recalling memories from the practice words participants were given feedback on the vividness of their description and then proceeded with the actual task. The adapted AMT was aimed to activate positive schemas by providing participants with positive adjectives such as *proud* or *relaxed* and instructing them to recall and narrate in detail personal memories related to these adjectives (Moscovitch et al., 2024).

**Figure 1:**
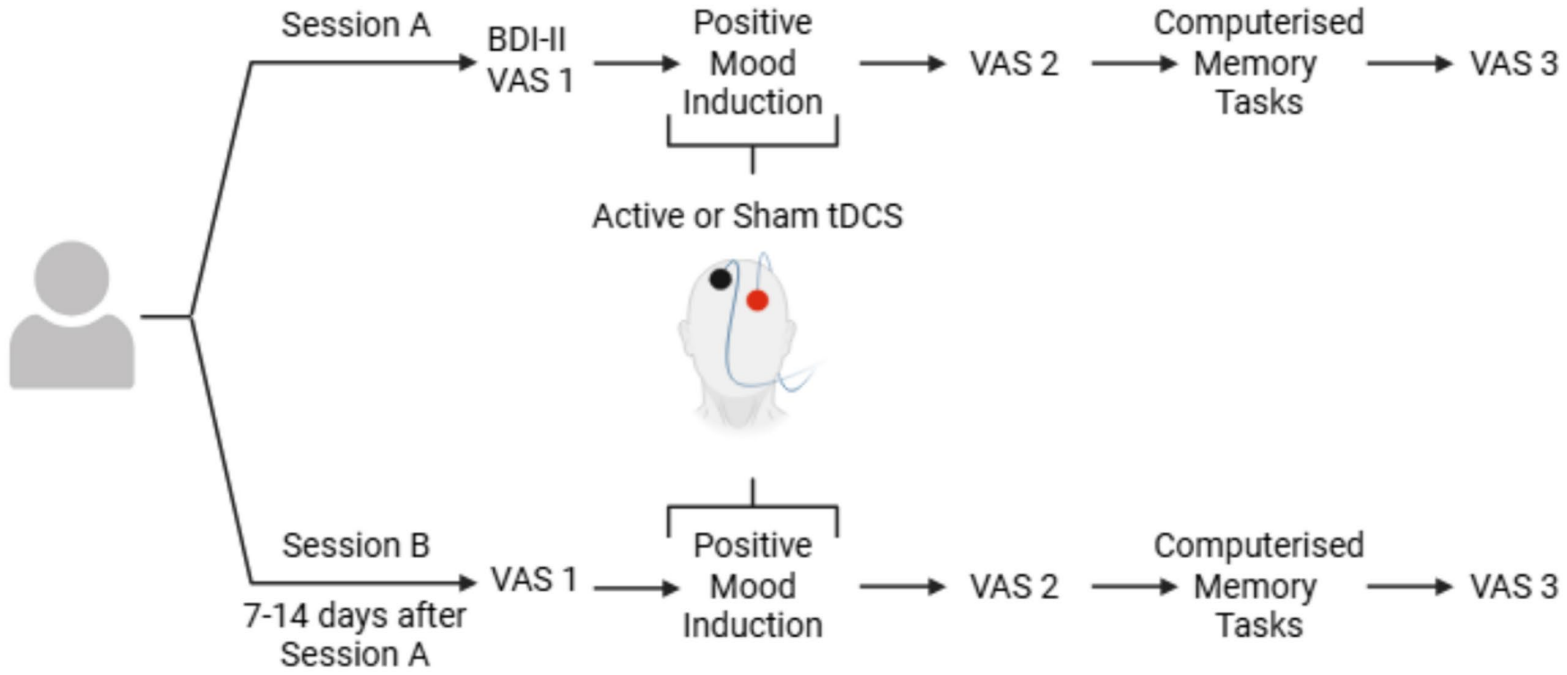
Graphical representation of the session outline. Each participant completed two sessions: one with active and one with sham tDCS. During the stimulation, they went through positive MIP including listening to 5 min of positive music and a positive version of the AMP. In each session, participants filled in the VAS at the same three timepoints as well as two computerised memory tasks. Each task had two versions; one for each session, the order of which was randomised.

Participants’ mood assessment scores were analysed and compared in two pairs: *Sad* VS *Happy* and *Stressed* VS *Relaxed* using the Visual Analogue Scale (VAS). This was completed before active or sham tDCS stimulation (VAS1), immediately after stimulation (VAS2), and at the end of the session (VAS3). *Stressed* and *Relaxed* were added alongside *Sad* and *Happy* to include a wider spectrum of emotional responses (see Figure 1).

### Memory tasks

Following the MIP and the active- or sham tDCS, participants completed two emotional memory tasks: the Deese–Roediger–McDermott False Memory Task (DRM Task, Appendix 2) and the Self-Referent Encoding Task (SRET; Derry and Kuiper, 1981, Appendix 3). A computerised version of each task was used to minimise experimenter biases.

### Deese–Roediger–McDermott false memory task

The computerised DRM task was used to measure implicit memory bias and included three stages: encoding, recall, and recognition. In the encoding phase, participants viewed 5 positive, 5 negative, and 5 neutral-themed lists of 10 semantically related words each, with each list corresponding to an unpresented critical lure. Following was the recall part, where participants freely listed the words that they remembered from the encoding phase, and rated their certainty for each. In the recognition part of the task, they judged whether each presented word was old or new, indicating their certainty. Prior to the encoding and recall phases, participants were offered the option to perform a practice version of the task so that they could familiarize themselves with the task. In the practice version, instead of stimulus words, participants saw animal names and were instructed to only focus on how the task works and not on the practice words. Key outcomes of the DRM task included recall accuracy, recognition rates (hit, false alarm, and critical lure), and a discrimination index (d-prime) representing sensitivity to falsely recognising lures.

### Self-referent encoding task

The computerised SRET assessed participants’ explicit memory bias for positive and negative verbal stimuli (Dobson and Shaw, 1987). Participants started with an encoding phase in which they viewed 12 positive and 12 negative adjectives one by one, each presented for 5 s, after which they specified whether they felt the adjective describes them (endorsement). After the encoding phase, a number-oriented, non-verbal distraction task was presented for 2 min. Following this task participants entered the retrieval phase, where they had 3 min to recall and enter as many words as possible of the previously presented adjectives. Endorsement and Recall scores were determined by dividing the number of endorsed/recalled adjectives within a specific valence category (positive or negative) by the total number of presented adjectives.

## Results

### No significant interaction between condition and time for the mood states

**Figure 2:**
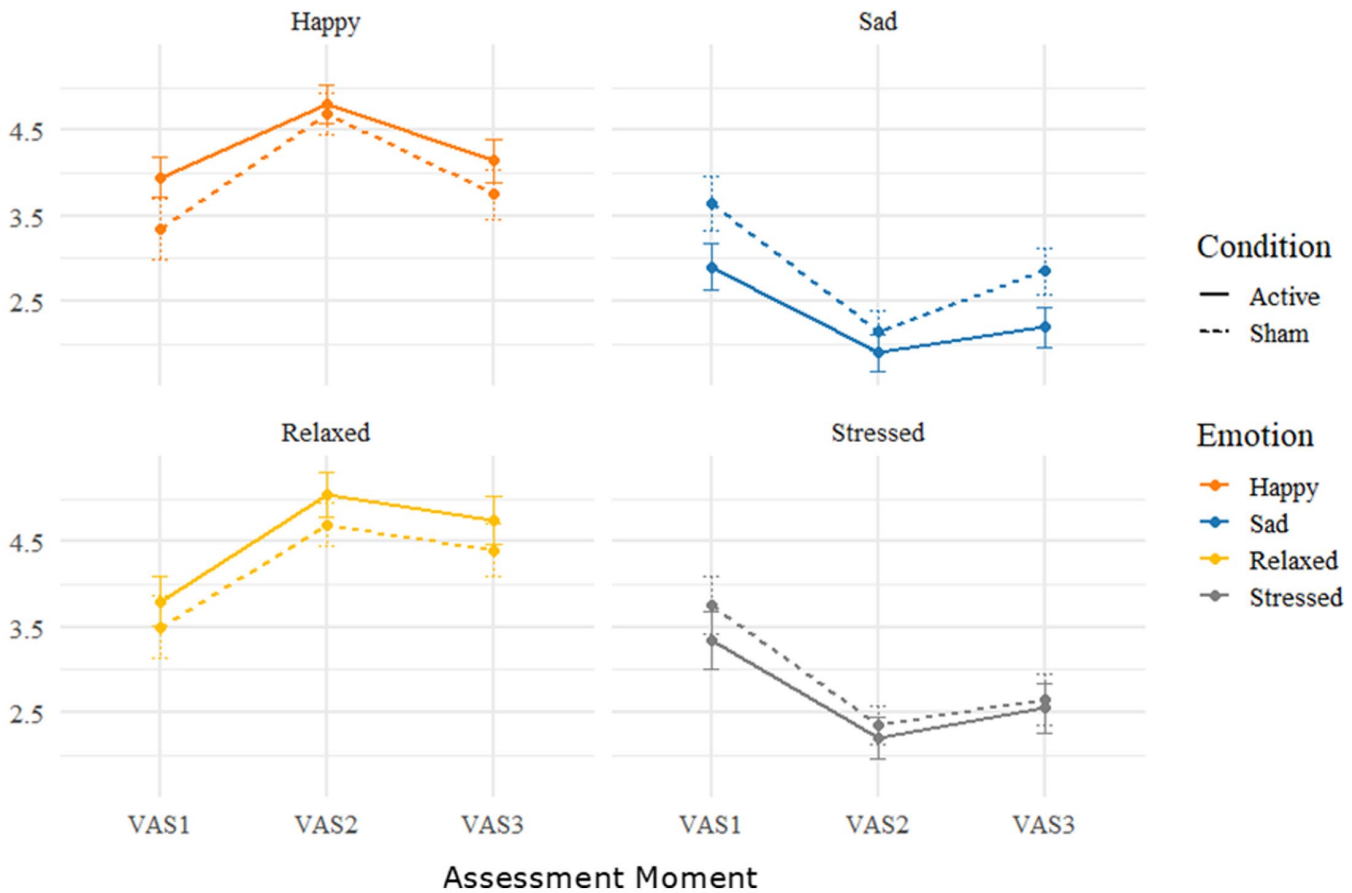
Results of the VAS assessing participants’ mood throughout the active tDCS and sham sessions. VAS1 was recorded at the start of the session, VAS2 directly after the MI, and VAS3 at the end of the session. Y-scale represents the reported score on VAS for each emotional state on a scale from 0 to 5. Error bars represent the standard error of the mean.

The interaction between condition (active tDCS, sham tDCS) and time (VAS1, VAS2, VAS3) for mood assessment scores was not significant for any of the mood states [sad *F*(2,18) = 1.237 *p* = 0.314 ηp^2^ = 0.121, happy *F*(2,18) = 1.001 *p* = 0.387 ηp^2^ = 0.067, stressed *F*(2,18) = 0.210 *p* = 0.812 *η_p_^2^* = 0.016 and relaxed *F*(2,18) = 0.008 *p* = 0.992 *η_p_^2^* < 0.001]. While the interaction between condition and time was not significant, given this was a pilot study aimed at informing future research and given the medium-to-large interaction effect on sadness, we explored *post hoc* pairwise comparisons. It was observed that at VAS3, participants in the active tDCS condition reported significantly less sadness (*N* = 20, *t* = 2.69, *p* = 0.008) than in the sham tDCS condition suggesting a possible mood induction enhancing effect of tDCS resulting in a maintained decrease in negative mood. This effect was not observed in any of the other mood state measurements (see Figure 2), with the all the pairwise group comparisons being non-significant (lowest *p* > 0.059).

### tDCS intervention results in weaker negative bias

**Figure 3:**
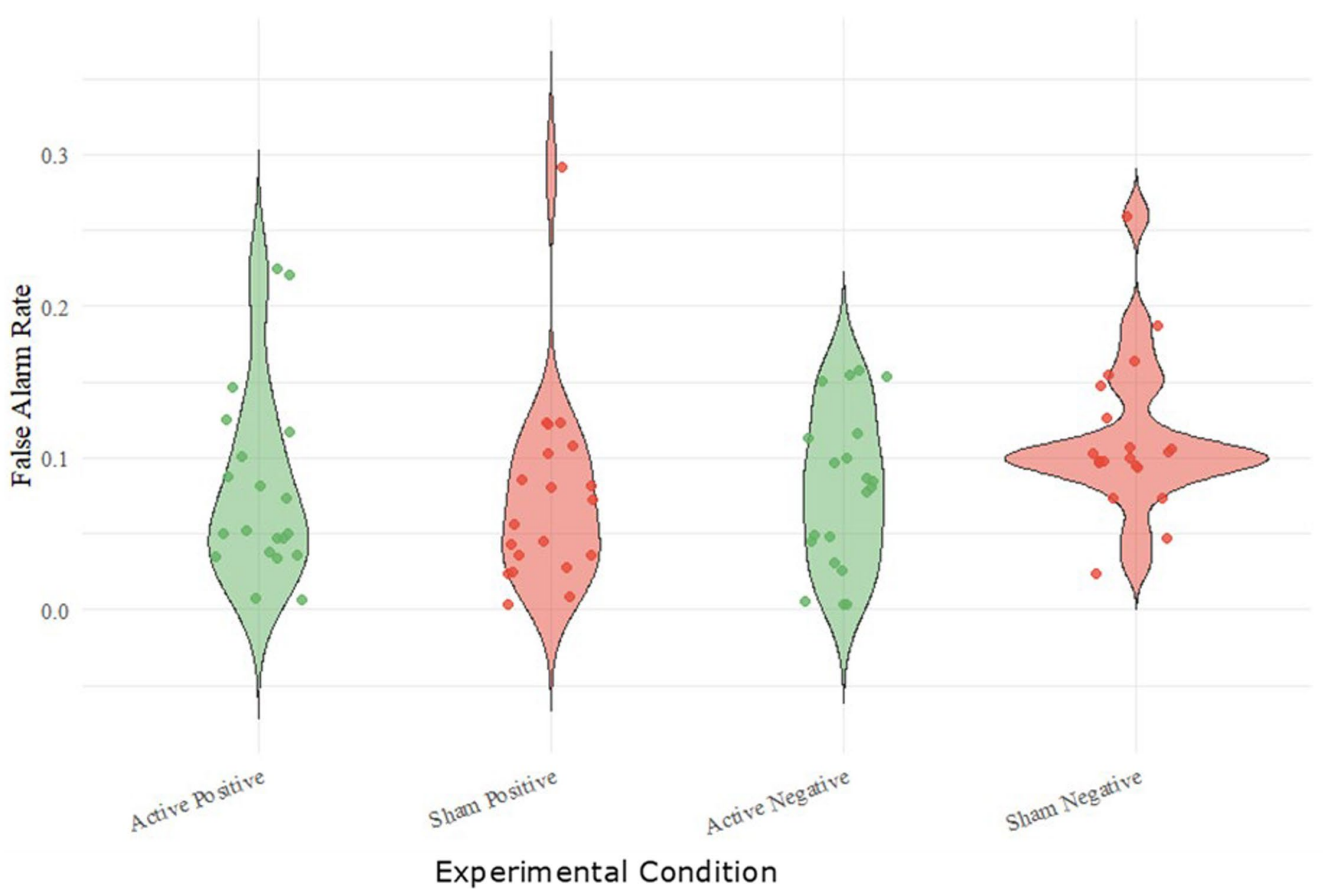
Ability of participants to discriminate between known and unknown positive or negative words (false alarm rate) in the active and sham tDCS condition during the recognition phase of the DRMFM task.

Data from one participant was excluded from the SRET-analysis because the participant reported misunderstanding the instructions (i.e., recalled the DRM task words; *N* = 19). No significant effects involving stimulation condition or session order were observed, indicating no evidence for carry-over effects (see tables in Appendices 4–6). In the DRM Task, there was a significant interaction between the condition and emotional valence of words [ANOVA: *F*(1,19) = 5.098, *p* = 0.036, *η_p_^2^* = 0.212; Appendix 4] on the tendency of participants to incorrectly recognise non-presented words (False Alarm Rate, Figure 3). Even though there was no significant difference between the two conditions for positive words [*N* = 20, one-tailed, *t* = −0.22, *p* = 0.415, *Cohen’s d* = −0.049, 95% CI (−0.212, 0.172)], participants who received anodal stimulation via tDCS falsely recognised significantly less negative words compared to the sham condition [*N* = 20, one-tailed*, t* = 2.68, *p* = 0.007, *Cohen’s d* = 0.599, 95% CI (−021, 0.175)], thus suggesting an effect of the stimulation in decreasing negative bias. Using the interquartile range method, the compared *t*-test results between sham and active tDCS were compared. These did not show significant difference between the sham and active conditions, with *p*-values for the original and cleaned datasets being 0.2374 and 0.6087, respectively. This pattern of effect, however, was not found for explicit memory bias measured using the SRET since there was no significant interaction between the stimulation condition and emotional valence of words [ANOVA, *F*(1,18) = 2.463, *p* = 0.134; Appendix 5] for the bias scores. While there was no significant interaction observed, there was a strong effect of valence in the SRET bias scores, with the average for positive terms (*N* = 19, *M* = 0.226, *SME* = 0.011) being significantly higher [*N* = 19, one-tailed, *t* = 5.88, *p* < 0.001, *Cohen’s d* = 1.348, 95% CI (−0.158, 0.006)], compared to negative terms (*N* = 19, *M* = 0.099, *SME* = 0.013).

### Active tDCS intervention increases memory accuracy

**Figure 4:**
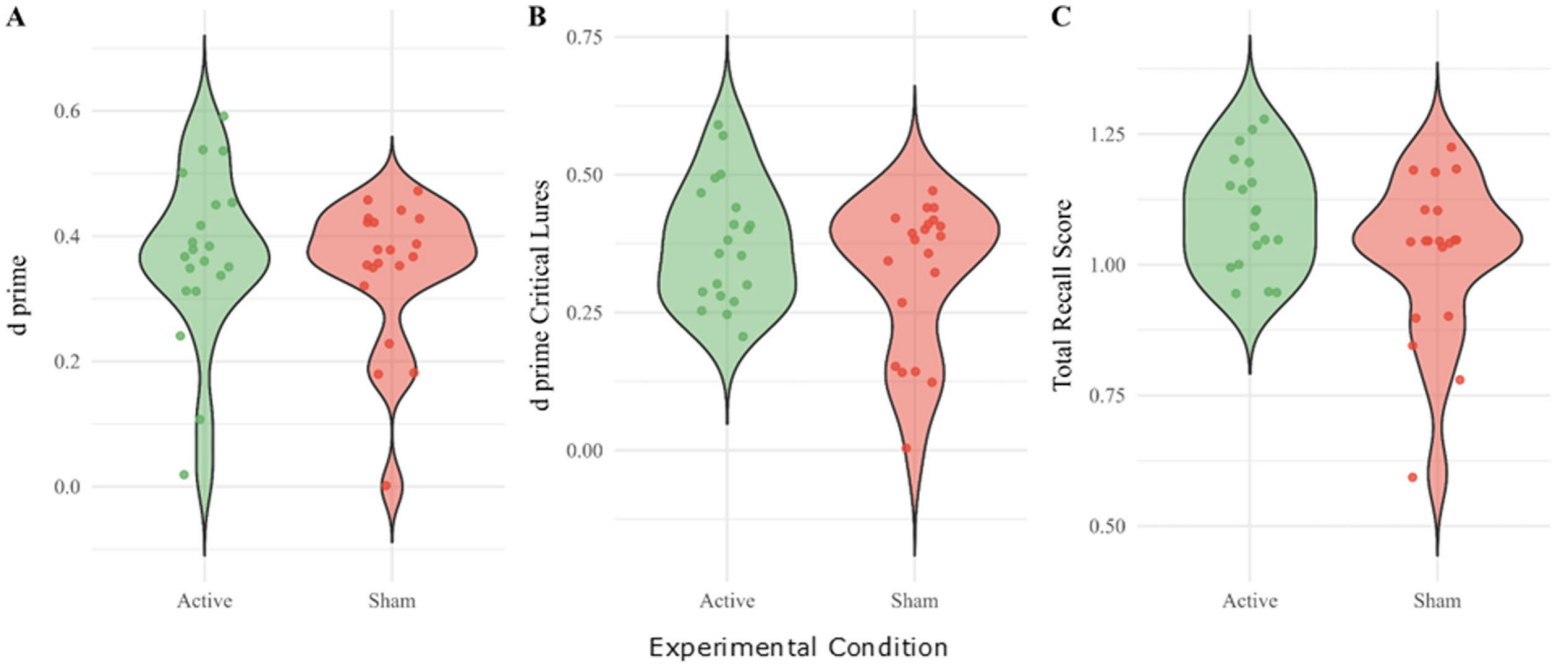
Measures for memory accuracy and performance in the active and sham tDCS condition. **(A)** Ability to discriminate between known and unknown terms in the DRM task. **(B)** Ability to identify lures in the DRM task. **(C)** Total correctly recalled words in the SRET.

In the recognition part of the DRM Task, the ability to discriminate between words that were seen in the encoding part (known) and those that were not (unknown) was estimated by the discrimination index (*d’*). Regarding the discrimination between all known and unknown terms, there was no significant difference between the two conditions as is shown in Figure 4 [*N* = 20, one-tailed, *t* = −1.40, *p* = 0.090, Cohen’s *d* = −0.312, 95% CI (−0.617, 0.123)]. However, when looking at the discrimination index only for critical lures, participants in the active tDCS condition had a significantly higher discrimination index compared to the sham tDCS condition [*N* = 20, one-tailed, *t* = −1.80, *p* = 0.044, Cohen’s *d* = −0.402 95% CI (−0.818, 0.061)]. Considering that the critical lures were specifically added to confuse the participants, the resilience of the active group in not being susceptible to the lure could indicate that the tDCS intervention increases memory accuracy.

Regarding overall memory performance, during the SRET task, participants in the active tDCS condition recalled significantly more words in total than those in the sham tDCS condition (see Figure 3; *N* = 19, one-tailed, *t* = −2.26, *p* = 0.018, Cohen’s *d* = −0.519). On the DRM Task, however, there was no significant interaction between condition (active or sham tDCS) and valence positive or negative term in the hit rate (correctly indicating that a known term was presented in the encoding task; ANOVA, *F*(1,19) = 0.390, *p* = 0.540 *η_p_*^2^ = 0.020; Appendix 6).

## Discussion

The present pilot study examined the effect of a positive personalised MIP on emotional schema-based memory and (duration of) mood changes. The tentative findings indicated that combining tDCS with MIPs may be associated with differences in mood-congruent false memory, including trends towards more positive false recall and reduced negative false recognition relative to sham stimulation. Although the interaction effects of condition and time were non-significant, the *post-hoc* pairwise comparisons suggested that participants in the active tDCS condition were less sad compared to the sham tDCS condition. Despite the small sample size, this pilot study offers preliminary insights into how tDCS may interact with personalised positive multimodal MIP, while highlighting the need for further investigation.

The repeated mood assessments showed that the MIP were associated with maintained mood changes, suggest that such combined and personalised procedures can achieve the temporal stability long sought after in MIPs research (Gillies and Dozois, 2021; Fernández-Pérez et al., 2022). The mood effect lingered long enough to still be present during the subsequent experimental tasks, which is notable given that singular, non-personalised MIPs typically produce only brief effects (Joseph et al., 2020; Gillies and Dozois, 2021). Integrating tDCS to augment the MIP effect may have contributed to prolonging the induced mood. The non-significant but moderate effect of the MIP on sadness (*η_p_*^2^ = 0.121) indicates an effect size that may inform future power calculations, though a larger sample would be necessary to detect this effect with sufficient power. Similarly, small-to-medium effects for happiness (*η_p_*^2^ = 0.067) warrant further investigation. The pattern of results is broadly consistent with the theorised working mechanisms of tDCS and other neuromodulation studies that found a moderate effect of single tDCS application on mood (Hu et al., 2024; Morgan et al., 2014; Stagg and Nitsche, 2011). The proposed ability of tDCS to prolong MIPs effects highlights the underlying role of schemas in mood, consistent with cognitive theories (Beck and Haigh, 2014; Bower, 1981; Disner et al., 2011). The primary aim of such procedures is to activate affectively valanced schemas, which in turn support the maintenance of the induced mood state. Our findings can guide hypotheses that a multimodal, self-referential MIPs approach supplemented by neuromodulation might be able to activate relevant emotional schemas long enough to influence both mood regulation and memory processing.

The neuromodulating effect of tDCS on memory was found for negative memory on the DRM Task as participants were significantly less likely to recognise negative words in the active tDCS condition than in the sham tDCS condition. This finding is consistent with cognitive models (Beck and Haigh, 2014) where activation of positive self-schemas enhanced by tDCS yield competition with negative schemas and in turn results in weakened negative memory bias. Within a dysphoric sample—typically resistant to cognitive change and where false memories are prominent and difficult to manipulate—this is an encouraging sign that even subtle modulation of negative schemas may be achievable in populations (Jobson et al., 2023; Joormann et al., 2009; Joormann et al., 2009; Otgaar et al., 2017). Our findings additionally tentatively support the notion that tDCS may enhance positive MIP through modulation of DL(PFC)-related cognitive regulation processes (Bovy et al., 2020; Brunoni et al., 2014; Morgan et al., 2014).

The enhancing effect of tDCS was not found for explicit memory bias on the SRET. Explicit memory biases may be more resistant to single-session modulation than implicit biases (Disner et al., 2017; O’Connor et al., 2021). The single tDCS session may therefore not have been sufficient to produce sufficient change. Importantly, during the SRET participants learn new words and reproduce them shortly thereafter (Dobson and Shaw, 1987). A second explanation for the lack of effect between tDCS and explicit memory bias is the strong baseline preference for positive information observed in the SRET. As is often the case with computerised memory bias tasks, a ceiling effect may have limited the sensitivity of the positive encoding phase, reducing the likelihood of detecting additional effects from the stimulation (Burden et al., 2021).

Although no significant effect was found when comparing the active and sham tDCS conditions on valanced memory, participants were significantly more able to distinguish critical lures from other stimuli in the active tDCS condition (*t* = −1.802, *p* = 0.044, η_p_^2^ = 0.039) than in the sham tDCS condition. The ability to recognise false cues is referred to in the literature as memory accuracy, a function in which the DLPFC plays an important role and can be influenced by tDCS (Berkers et al., 2017; Boggio et al., 2009; Meléndez et al., 2021). Other research using transcranial magnetic stimulation on the medial PFC showed also reduced false recall of critical lures (Berkers et al., 2017). Overall, the effects of tDCS on memory performance enhancement in our experiment were inconclusive, with no significant enhancement found across either computer tasks or other relevant readouts. This is in contrast to previous studies (Brunoni et al., 2014; Hill et al., 2016; Javadi et al., 2012; Lv et al., 2024; Mancuso et al., 2016; Otgaar et al., 2017; Schwippel et al., 2025), although similar null findings have also been reported (Burden et al., 2021; Mainz et al., 2020). We hypothesised that repeated tDCS sessions would be required to produce reliable memory enhancement effects.

The results of the present study highlight some important trends, but the non-significant effects and results not in-line with our hypotheses should not be overlooked. There are several reasons that could explain these non-significant results, the most prominent being the small sample size and singular tDCS session. Moreover, the cross-over design of the present study cannot rule out the possibility of carry-over or practice effects. Nonetheless, the one-week washout period between sessions, combined with the counterbalanced design, likely reduced the risk of such confounds. Psychoactive medication use may have influenced our participants’ emotional state and cognitive functioning, while limited differential influence on the conditions can be expected in this within-subject design. Finally, as experimenters were not blinded to stimulation condition when administering the memory tasks, this may have introduced expectancy effects. Future studies could move beyond controlled laboratory settings by integrating repeated or longer at-home tDCS sessions (Charvet et al., 2020) with personally tailored MIPs (Tutunji et al., 2024; Vrijsen et al., 2021). Such combinations may enhance the potential of tDCS to facilitate schema change. This pilot study explores whether combining a multimodal, positive MIPs with tDCS can influence mood and impact schema-related memory processes in dysphoric individuals. Taken together, the findings should be interpreted cautiously, as the study was exploratory in nature, with a small sample size and predominantly non-significant effects. Beyond demonstrating the feasibility of targeting the DLPFC with tDCS and the added value of supplementing MIPs with personally relevant stimuli, the findings tentatively indicate the relevance of schema-focused interventions in future clinical research on depressed patients.

## Data Availability

The datasets presented in this study can be found in online repositories. The data supporting the findings are archived in the Radboud Data Repository.
