# Supplementary material for: Enhancing positive memory schemas with tDCS: a pilot study
Source: Front Hum Neurosci. 2026 Mar 16;20:1722485. doi: 10.3389/fnhum.2026.1722485 (PMC13034138; doi:10.3389/fnhum.2026.1722485)
Supplement: Supplementary file 1 [file Table_1.docx]

**Appendix**

*Appendix 1: Autobiographical Memory Task word list per version including translation in English*

|  | **Version 1** | **Version 2** |
| --- | --- | --- |
|  | Gelukkig (Fortunate) | Ontspannen (Relaxed) |
|  | Veilig (Safe) | Trots (Proud) |
|  | Belangstellend (Interested) | Prettig (Pleasant) |
|  | Succesvol (Successful) | Zorgeloos (Carefree) |
|  | Verrast (Surprised) | Sympathiek (Sympathetic) |
| **Practice words** | Vriendelijk (Friendly)  Respectvol (Respectful) | Genieten (Enjoyable)  Mooi (Pretty) |

*Appendix 2: Deese–Roediger–McDermott False Memory Task word list per version*

***Version 1***

*Encoding Phase, Negative*

| **Critical Lure** | **Encoded Words** | **Translation of Encoded Words** |
| --- | --- | --- |
| Kanker (Cancer) | Ziekte, Chemo, Tumor, Bestraling, Behandeling, Kaal, Long, Borst, Scheldwoord, Roken | Illness, Chemotherapy, Tumor, Radiation, Treatment, Bald, Lung, Breast, Swear word, Smoking |
| Ongeluk (Misfortune) | Ongeval, Aanrijding, Botsing, Pech, Verkeer, Gewond, Kapot, File, Tegenslag, Ramp | Accident, Collision, Crash, Bad luck, Traffic, Injured, Broken, Traffic jam, Setback, Disaster |
| Ruzie (Argument) | Onenigheid, Dispuut, Aanvaring, Onvrede, Rel, Stampei, Gevecht, Opschudding, Bonje, Discussie | Disagreement, Dispute, Confrontation, Discontent, Quarrel, Commotion, Fight, Uproar, Squabble, Discussion |
| Alleen (Alone) | Enig, Eenzaam, Afgezonderd, Isolement, Niemand, Stil, Weggaan, Uitgestorven, Leeg  Verlaten | Only, Lonely, Isolated, Isolation, Nobody, Quiet, Leaving, Extinct, Empty, Abandoned |
| Spijt (Regret) | Schuldgevoel, Geweten, Verkeerd, Sorry, Fout, Schaamte, Excuses, Onrecht, Berouw, Inkeer | Guild, Conscience, Wrong, Sorry, Error/Fault, Shame, Apologies, Injustice, Remorse, Repentance |

*Encoding Phase, Positive*

| **Critical Lure** | **Encoded Words** | **Translation of Encoded Words** |
| --- | --- | --- |
| Vrijheid (Freedom) | Onafhankelijkheid, Democratie, Keuzes, Mensenrechten, Gelijkheid, Zelfstandigheid, Nederland, Autonomie, Bevrijdingsdag, Ongedwongen | Independence, Democracy, Choices, Human rights, Equality, Self-reliance, The Netherlands, Autonomy, Liberation Day, Unconstrained |
| Vacantie (Vacation) | Zon, Ontspannen, Strand, Buitenland, Bijkomen, Zee, Avontuur, Camping, Cocktail, Hotel | Sun, Relax, Beach, Abroad, Recuperate/Rest, Sea, Adventure, Camping, Cocktail, Hotel |
| Warmte (Warmth) | Hitte, Verwarming, Deken, Openhaard, Vuur, Heet, Gloed, Douche, Temperatuur, Kachel | Heat, Heating, Blanket, Fireplace, Fire, Hot, Glow, Shower, Temperature, Heater |
| Trowen (Wedding) | Ring, Jurk, Ja-woord, Verbintenis, Taart, Bruid, Aanzoek, Bruidegom, Bloemen, Pak | Ring, Dress, “I do”, Commitment, Cake, Bride, Proposal, Groom, Flowers, Suit |
| Liefde (Love) | Houden van, Romantiek, Verliefd, Passie, Zoenen, Affectie, Vlinders, Valentijnsdag, Rood, Cupido | To love, Romance, In love, Passion, Kissing, Affection, Butterflies (feeling), Valentine’s Day, Red, Cupid |

*Encoding Phase, Neutral*

| **Critical Lure** | **Encoded Words** | **Translation of Encoded Words** |
| --- | --- | --- |
| Auto (Car) | Truck, Bus, Trein, Benzine, Voertuig, Rijden, Jeep, Ford, Race, Sleutels | Truck, Bus, Train, Gasoline/Petrol, Vehicle, Drive, Jeep, Ford, Race, Keys |
| Ruiken (Smell) | Neus, Ademen, Snuiven, Aroma, Horen, Zien, Neusgat, Vleug, Reuk, Rieken | Nose, Breathe, Sniff, Aroma, Hear, See, Nostril, Wing, Smell (noun), Smell (verb) |
| Langzaam (Slow) | Snel, Traag, Stop, Sloom, Slak, Behoedzaam, Vertraging, Passief, Schildpad, Aarzelend | Fast, Slow, Stop, Sluggish, Snail, Cautious, Delay, Passive, Turtle, Hesitant |
| Stoel (Chair) | Tafel, Zitten, Poot, Zitplek, Bank, Bureau, Zetel, Fauteuil, Kruk, Troon | Table, Sit, Leg (of furniture), Seat, Couch/Sofa, Desk, Seat/Armchair, Armchair, Stool, Throne |
| Berg (Mountain) | Heuvel, Vallei, Klimmen, Spits, Top, Alpen, Piek, Vlakte, Gletsjer, Geit | Hill, Valley, Climb, Peak, Summit, Alps, Peak, Plain, Glacier, Goat |

*Recognition Phase, Additional words*

| **Category** | **Additional Recognition Words** | **Translation** |
| --- | --- | --- |
| Negative | Tragisch, Wantrouwend, Vijand, Steekpartij, Nerveus, Onrust, Fataal, Intimidatie, Bedrieglijk, Verslaafd, Marteling, Psychopaat, Gijzelaar, Afkeer, Slachting | Tragic, Distrustful, Enemy, Stabbing, Nervous, Restless, Fatal, Intimidation, Deceitful, Addicted, Torture, Psychopath, Hostage, Aversion, Massacre |
| Positive | Schattig, Gezellig, Eerlijk, Ideaal, Aardig, Wijsheid, Positief, Vastberaden, Briljant, Volmaakt, Avontuurlijk, Genezen, Interessant, Succes, Attent | Cute, Cozy, Honest, Ideal, Kind, Wisdom, Positive, Determined, Brilliant, Perfect, Adventurous, Healed, Interesting, Success, Considerate |
| Neutral | Macht, Gewoonte, Ijdelheid, Gemiddeld, Maand, Verbaasd, Normaal, Materiaal, Aanbod, Bezoek, Geslacht, Tijd, Kieskeurig, Baan, Volgzaam | Power, Habit, Vanity, Average, Month, Surprised, Normal, Material, Offer, Visit, Gender, Time, Picky, Job, Obedient |

***Version 2***

*Encoding Phase, Negative*

| **Critical Lure** | **Encoded Words** | **Translation of Encoded Words** |
| --- | --- | --- |
| Dief (Thief) | Indringer, Ongewenst, Stelen, Inbreker, Misdaad, Rover, Boef, Schurk, Bivakmuts | Intruder, Unwanted, Steal, Burglar, Crime, Robber, Thief, Villain, Balaclava |
| Boos (Anger) | Kwaad, Razernij, Laaiend, Opvliegend, Aanvallen, Schoppen, Humeur, Schreeuwen, Schelden, Haat | Angry, Rage, Fiery, Short-tempered, Attack, Kick, Mood, Shouting, Swearing, Hatred |
| Gemeen (Mean) | Wreed, Meedogenloos, Harteloos, Kwaadaardig, Geniepig, Grof, Onaardig, Vals, Hatelijk, Cru | Cruel, Merciless, Heartless, Malicious, Sneaky, Rude, Unkind, False, Hateful, Cruel |
| Oorlog (War) | Soldaat, Wapens, Leger, Strijder, Bommen, Kamp, Vluchten, Militairen, Tanks, Duitsland | Soldier, Weapons, Army, Fighter, Bombs, Camp, Flee, Military, Tanks, Germany |
| Dood (Dead) | Overlijden, Verdriet, Rouwen, Begrafenis, Crematie, Verlies, Doodskist, Oud, Gestorven, Zwart | Death, Sorrow, Mourning, Funeral, Cremation, Loss, Coffin, Old, Deceased, Black |

*Encoding Phase, Positive*

| **Critical Lure** | **Encoded Words** | **Translation of Encoded Words** |
| --- | --- | --- |
| Geboorte (Birth) | Baby, Bevalling, Zwanger, Moeder, Baring, Vader, Beschuit, Verloskundige, Nieuw, Navelstreng | Baby, Birth, Pregnant, Mother, Delivery, Father, Biscuit, Midwife, New, Umbilical cord |
| Muziek (Music) | Noot, Geluid, Piano, Zingen, Radio, Festival, Melodie, Concert, Instrument, Ritme | Note, Sound, Piano, Singing, Radio, Festival, Melody, Concert, Instrument, Rhythm |
| Blij (Happy) | Vrolijk, Plezier, Grappig, Opgewekt, Gniffelen, Grijns, Leuk, Speels, Gelukkig, Opgetogen | Cheerful, Fun, Funny, Cheerful/Upbeat, Giggle, Grin, Nice, Playful, Happy, Excited |
| Vriend (Friend) | Kameraad, Band, Broeder, Makker, Bondgenoot, Kompaan, Delen, Buddy, Collega, Maat | Comrade, Band, Brother, Mate, Ally, Companion, Share, Buddy, Colleague, Friend |
| Vrede (Freedom) | Vreedzaam, Harmonie, Eenheid, Wapenstilstand, Kalmte, Duif, Verdrag, Overeenkomst, Rust, Wit | Peaceful, Harmony, Unity, Armistice, Calm, Dove, Treaty, Agreement, Rest, White |

*Encoding Phase, Neutral*

| **Critical Lure** | **Encoded Words** | **Translation of Encoded Words** |
| --- | --- | --- |
| Stad (City) | Burgemeester, Menigte, Provincie, Gemeente, Straat, Dorp, Platteland, Amsterdam, Plein, Park | Mayor, Crowd, Province, Municipality, Street, Village, Countryside, Amsterdam, Square, Park |
| Rivier (River) | Water, Beek, Meer, Maas, Boot, Getijde, Zwemmen, Stroom, Sloot, Schip | Water, Stream, Lake, Maas (River), Boat, Tide, Swimming, Current, Ditch, Ship |
| Brood (Bread) | Boter, Voedsel, Eten, Boterham, Rogge, Jam, Melk, Bloem, Kaas, Deeg | Butter, Food, Eat, Sandwich, Rye, Jam, Milk, Flour, Cheese, Dough |
| Raam (Window) | Deur, Glas, Ruit, Schaduw, Venster, Lijst, Lappen, Open, Gordijn, Uitzicht | Door, Glass, Pane, Shade, Window, Frame, Cloth, Open, Curtain, View |
| Slapen (Sleep) | Bed, Wekker, Wakker, Moe, Droom, Waken, Dommelen, Woelen, Maffen, Pitten | Bed, Alarm Clock, Awake, Tired, Dream, Stay Awake, Doze, Toss and Turn, Nap, Sleep |

*Recognition Phase, Additional words*

| **Category** | **Additional Recognition Words** | **Translation** |
| --- | --- | --- |
| Negative | Overbelasting, Verslaving, Zondig, Slapeloosheid, Furieus, Corrupt, Hartstilstand, Misbruik, Omkopen, Gevaar, Ontvoering, Pessimist, Radeloos, Aanslag, Trauma | Overload, Addiction, Sinful, Insomnia, Furious, Corrupt, Cardiac Arrest, Abuse, Bribe, Danger, Kidnapping, Pessimist, Desperate, Attack, Trauma |
| Positive | Verrassing, Schoonheid, Dankbaar, Warmhartig, Sympathie, Compliment, Prachtig, Zelfrespect, Fantasie, Fris, Comfort, Ontdekken, Intelligent, Vitaal, Held | Surprise, Beauty, Grateful, Warm-hearted, Sympathy, Compliment, Beautiful, Self-respect, Imagination, Fresh, Comfort, Discover, Intelligent, Vital, Hero |
| Neutral | Inhoud, Fruit, Kaars, Frequentie, Soort, Kordaat, Schedel, Kluis, Ogenblik, Proces, Controleren, Vinger, Boodschap, Draaien, Handelaar | Content, Fruit, Candle, Frequency, Type, Decisive, Skull, Safe, Moment, Process, Check/Control, Finger, Message, Turn, Trader |

*Appendix 3:* *Self-Referent Encoding Task word list*

| **Version 1**  **(Original)** | **Version 1 (Translation)** | **Version 1**  **(Original)** | **Version 2 (Translation)** |
| --- | --- | --- | --- |
| sBlij | Happy | Ruimdenkend | Open-minded |
| Afgunstig | Envious | Afstotelijk | Disgusting |
| Succesvol | Successful | Liefdevol | Loving |
| Romantisch | Romantic | Geduldig | Patient |
| Gemeen | Mean | Instabiel | Unstable |
| Verdrietig | Sad | Asociaal | Asocial |
| Geïnteresseerd | Interested | Tevreden | Content |
| Minderwaardig | Inferior | Afgewezen | Rejected |
| Leuk | Fun | Empathisch | Empathetic |
| Moedig | Brave | Opgewekt | Cheerful |
| Boos | Angry | Kwetsbaar | Vulnerable |
| Aantrekkelijk | Attractive | Aardig | Kind |
| Nutteloos | Useless | Woedend | Furious |
| Hoopvol | Hopeful | Eerlijk | Honest |
| Dom | Stupid | Bang | Afraid |
| Gezellig | Cozy | Levendig | Lively |
| Pessimistisch | Pessimistic | Negatief | Negative |
| Afhankelijk | Dependent | Harteloos | Heartless |
| Tolerant | Tolerant | Optimistisch | Optimistic |
| Gespannen | Tense | Prikkelbaar | Irritable |
| Betrouwbaar | Reliable | Behulpzaam | Helpful |
| Kwaadaardig | Malicious | Ongelukkig | Unhappy |
| Enthousiast | Enthusiastic | Getalenteerd | Talented |
| Saai | Boring | Neerslachtig | Gloomy |

*Appendix 4:*

*ANOVA table false alarm rate condition and emotional valence of words DRM*

| **Effect** | **df** | **F** | **p** | **MSE** | **ηp²** |
| --- | --- | --- | --- | --- | --- |
| Condition | 1, 19 | 2.25 | .150 | .002 | .106 |
| Valence | 1, 19 | 5.08 | .036 | .002 | .211 |
| Condition × Valence | 1, 19 | 5.10 | .036 | .001 | .212 |

*Note.* Greenhouse–Geisser corrected statistics are identical to sphericity-assumed values because all within-subject factors had two levels and therefore did not violate the sphericity assumption. Partial eta squared (ηp²) is reported as the effect size.

*ANOVA table false alarm rate condition and emotional valence of words DRM including Order as between subject*

| **Effect** | **df** | **F** | **p** | **ηp²** |
| --- | --- | --- | --- | --- |
| Condition | 1, 18 | 2.14 | .161 | .106 |
| Condition × Order | 1, 18 | 0.02 | .888 | .001 |
| Valence | 1, 18 | 4.83 | .041 | .212 |
| Valence × Order | 1, 18 | 0.05 | .824 | .003 |
| Condition × Valence | 1, 18 | 4.86 | .041 | .212 |
| Condition × Valence × Order | 1, 18 | 0.10 | .760 | .005 |

*Appendix 5:*

*ANOVA table interaction stimulation condition and emotional valence of words SRET*

| **Effect** | **df** | **F** | **p** | **ηp²** |
| --- | --- | --- | --- | --- |
| Condition | 1, 18 | 2.16 | .159 | .107 |
| Valence | 1, 18 | 34.55 | < .001 | .657 |
| Condition × Valence | 1, 18 | 2.46 | .134 | .120 |

*Note.* Greenhouse–Geisser corrected statistics are identical to sphericity-assumed values because all within-subject factors had two levels and therefore did not violate the sphericity assumption. Partial eta squared (ηp²) is reported as the effect size.

*ANOVA table interaction stimulation condition and emotional valence of words SRET with Order (sham or active) as between subject*

| **Effect** | **Test** | **F** | **df (hyp, err)** | **p** | **ηp²** |
| --- | --- | --- | --- | --- | --- |
| Condition | Pillai’s Trace | 1.99 | 1, 17 | .176 | .105 |
| Condition × Order | Pillai’s Trace | 0.47 | 1, 17 | .500 | .027 |
| Valence | Pillai’s Trace | 32.61 | 1, 17 | < .001 | .657 |
| Valence × Order | Pillai’s Trace | 0.01 | 1, 17 | .927 | .001 |
| Condition × Valence | Pillai’s Trace | 2.40 | 1, 17 | .140 | .124 |
| Condition × Valence × Order | Pillai’s Trace | 0.13 | 1, 17 | .719 | .008 |

*Appendix 6:*

*ANOVA table condition (active or sham tDCS) and valence (positive or negative term in the hit rate) DRM*

| **Effect** | **df** | **F** | **p** | **MSE** | **ηp²** |
| --- | --- | --- | --- | --- | --- |
| Condition | 1, 19 | 0.13 | .725 | .002 | .007 |
| Valence | 1, 19 | 0.01 | .931 | .001 | .000 |
| Condition × Valence | 1, 19 | 0.39 | .540 | .001 | .020 |

*Note.* Greenhouse–Geisser corrected statistics are identical to sphericity-assumed values because all within-subject factors had two levels and therefore did not violate the sphericity assumption. Partial eta squared (ηp²) is reported as the effect size.

*ANOVA table condition (active or sham tDCS) and valence (positive or negative term in the hit rate) with Order (sham or active) as between subject in the DRM*

| **Effect** | **df** | **F** | **p** | **ηp²** |
| --- | --- | --- | --- | --- |
| Condition | 1, 17 | 1.99 | .176 | .105 |
| Condition × Order | 1, 17 | 0.47 | .500 | .027 |
| Valence | 1, 17 | 32.61 | < .001 | .657 |
| Valence × Order | 1, 17 | 0.01 | .927 | .001 |
| Condition × Valence | 1, 17 | 2.40 | .140 | .124 |
| Condition × Valence × Order | 1, 17 | 0.13 | .719 | .008 |
